# A comparison of five international clinical trial registers with the South African register for access to information and usability

**DOI:** 10.11604/pamj.2018.29.224.12683

**Published:** 2018-04-24

**Authors:** Mncengeli Sibanda, Robert Summers, Johanna Catharina Meyer

**Affiliations:** 1 School of Pharmacy, Sefako Makgatho Health Sciences University, Pretoria, South Africa

**Keywords:** Clinical trials, register, registry, declaration of Helsinki, good clinical practice, International conference of harmonisation, medicines control council, SANCTR

## Abstract

**Introduction:**

in November, 2005, the South African (SA) National Department of Health (NDoH) mandated that, as from the 1^st^ December, 2005, all new clinical trials to be conducted in the country must be registered on the South African National Clinical Trials Register (SANCTR). The objective was to compare access to the information contained in and the usability of the SANCTR with five other international on-line clinical trials registers.

**Methods:**

Access to SANCTR was determined through the use of three search engines using the keywords “South African Clinical Trials.” Five high-profile international registers were identified and accessed for comparative purposes. Each register was investigated for information on trials conducted in South Africa using a standardised data extraction form which listed 24 data items. The usability of the various on-line registers was determined through a self-administered questionnaire adapted from the five key usability factors previously defined in literature. Heuristic evaluation was carried out with 10 'experts' (Pharmacy staff and postgraduate students at Sefako Makgatho Health Sciences University (SMU)). Data generated from the heuristic evaluation were analysed using descriptive statistics, univariate and multivariate analyses.

**Results:**

The SANCTR website had the highest ranking for access amongst the registers in all three selected search-engines after an internet search using the keywords “South African Clinical Trials”. The total number of clinical trials registered varied among the registers. The WHO's International Clinical Trials Registry Platform (ICTRP) recorded 2 599 trials carried out in South Africa, with 2 260 registered in the ClinicalTrials.gov register, 2 196 in the SANCTR and 978, 149 and 174 in the European Union (EU), International Standard Randomised Controlled Trial Number (ISRCTN) and Pan African Clinical Trials (PACTR) registers respectively. The websites ClinicalTrials.gov and ISRCTN provided greater overall information per clinical trial registered and provided information on all 24 clinical trials data items. The PACTR had information on 23 of the 24 data items. The WHO and EU registers each contained 19 data items. The SANCTR provided the least information, only 11 data items. The heuristic evaluation identified ClinicalTrials.gov as the 'best' site, while the PACTR had the lowest rating for layout and design. The EU register and SANCTR were the least easily navigable. The respondents had the least satisfaction while using the 'Search' option in the SANCTR. Users also reported the SANCTR and the PACTR had the lowest overall user-friendliness.

**Conclusion:**

The fact that the SANCTR contains less information on SA clinical trials than other registers and is the least user-friendly warrants utmost attention. The study puts forward a case to the regulatory authority (currently the Medicines Control Council) as it takes on a new structure and working arrangements as the South African Health Products Regulatory Authority to optimise the SANCTR to be more user-friendly and contain more complete information on clinical trials conducted in SA.

## Introduction

In 2006 the South African National Department of Health (NDoH) issued revised guidelines for good practice in the conduct of clinical trials in human participants in South Africa to include a directive first issued in 2005, that all new clinical trials to be conducted in the country and which had received ethical approval must be registered on the South African National Clinical Trials Register (SANCTR) before participants are enrolled [1]. The call by the NDoH to establish the SANCTR was in response to the 2004 amendments to the Declaration of Helsinki, which stated that “Every clinical trial must be registered in a publicly accessible database before recruitment of the first subject” [2]. The International Committee of Medical Journal Editors (ICMJE) made a similar call with effect from 1 July, 2005 that all trials were required to be registered in a clinical trials register as a precondition for publication of the results in any of the worldwide peer-reviewed journals in its network [3]. The reasons for the establishment of clinical trials registers include the need to ensure that decisions about health care are informed by all available evidence and to safeguard transparency in the reporting of clinical trial results [4]. The information included in clinical trial registers permits knowledge-sharing. Knowledge-sharing enables researchers, health care workers and the public to be aware of recruiting for trials so as to facilitate ease of recruitment and peer review of clinical trial methodology to identify potential problems in the study design [5]. Additionally, the registration of clinical trials in registers allows for the potential for collaboration with other researchers through prospective meta-analysis, as well as to identify gaps in clinical trials research and minimise duplication [6]. Another reason for the establishment of clinical trial registers is to check and ensure that the results of trials are published [6]. It is envisaged that the SANCTR would provide users of the register with easily accessible and up to date information on all clinical trials on human participants conducted in the country. The register should provide information about a trial's objectives, eligibility criteria, location, and contact details of the trial's Principal Investigator [1].

According to the World Health Organization (WHO), the registration of all interventional trials is a scientific, ethical and moral responsibility [1]. The WHO prescribed a data set with 20 minimum data items which must be documented in the register for each clinical trial [7]. Worldwide, various clinical trial registers exist and are available per country (e.g. SANCTR) or per region (e.g. the European Union Clinical Trials Register (EUCTR) or per disease (e.g. AIDS info clinical trials). There are 24 clinical trial registers that are national, regional or international in scope [8]. Of these, 15 primary registers belong to the WHO register network [7]. The WHO Registry Network provides prospective investigators with a platform to exchange information and work together to establish best practice [7]. In order to be included in the WHO Registry Network, the register must meet specific criteria for quality and validity, accessibility, technical capacity and administration and contain 20 prescribed minimum data items [7]. Likewise, the ICMJE will only consider publishing reports of trials registered in a register which is part of the WHO Registry Network in any of the journals in its network [3]. Details of the SANCTR and five other international, widely-used clinical trial registers follow; the SANCTR is managed by the Medicines Control Council (MCC). All clinical trials to be conducted in South Africa are required to be registered on the SANCTR [1]. Clinical trial sponsors must register their proposed trial at http://www.saclinicaltrials.gov.za/ via a web-based data entry system called the “SANCTR Toolkit” [9]. Upon registration, each clinical trial is assigned a SANCTR number. The SANCTR does not meet the criteria for inclusion into the WHO Registry Network. South African trialists therefore have to register in both the SANCTR (to meet South African requirements) and a WHO recognised register in order to meet ICMJE requirements for publication [1,3]. The International Clinical Trials Registry Platform (ICTRP) system of the WHO is a network of international clinical trials registers to ensure a single point of access and the unambiguous identification of trials [10].

The WHO consists of 194 member states. However, only 15 registers covering 170 countries are incorporated into the meta-register on the ICTRP website [10]. The SANCTR is not listed as a member of the ICTRP although studies carried out in South Africa are listed in this register. ClinicalTrials.gov is an American-based register maintained by the United States of America (USA) National Library of Medicine (NLM) at the National Institutes of Health (NIH). It contains information on clinical trials in all 50 states in the USA as well as in 187 countries worldwide, including South Africa [11]. It is not recognised by the WHO as a primary register. It does however have a data sharing agreement with the WHO [10]. The International Standard Randomised Controlled Trial Number (ISRCTN) is a simple numeric system for the unique identification of clinical trials worldwide. The ISRCTN register is managed by the ISRCT Network and administered by BioMed Central, a publisher of open access peer-reviewed biomedical journals [12]. It contains information on clinical trials carried out worldwide (including SA). It is a primary register which is recognised by ICMJE and the WHO [3, 10]. The Pan African Clinical Trials Register (PACTR) is a regional register of clinical trials conducted in Africa. It is a subset of the WHO ICTRP Network [10]. Initially it was established as an AIDS, TB and Malaria clinical trials registry [13]. It also contains some information on CTs carried out in South Africa. It is a primary register which is recognised by ICMJE and the WHO [3, 10]. The European Union Clinical Trial Register (EUCTR) is a register of interventional clinical trials on medicines conducted in the EU or clinical trials conducted outside the EU/European Economic Area (EEA) [14]. It is also a primary register which is recognised by ICMJE and the WHO [3, 10]. Access to clinical trial registers has been enhanced through having them available through the World Wide Web. This allows potential users to access such registers easily via the respective websites. A website will be widely used if it is easy to find in the first instance [15].

Most websites, especially those with a commercial orientation, need a high ranking on a search engine for one or more keywords or phrases [15]. It is important for on-line clinical trial registers to be easy to use to allow users to access effortlessly the information they require. A website will be highly utilised if the design and layout are well-structured and easily navigable and if information on the webpage is easy to find [15]. The ISO 9241-11 standard defines usability as “The extent to which a product can be used by specified users to achieve specified goals with effectiveness, efficiency and satisfaction in a specified context of use” [16]. Nielsen (1993) grouped website usability in five key attributes: Learnability: the ease of learning the functionality and the behaviour of the system; Efficiency: the level of attainable productivity, once the user has learned the system; Memorability: the ease of remembering the system's functionality, so that the casual user can return to the system after a period of non-use, without needing to learn how to use it again; Few errors: the capability of the system to feature a low error rate, to support users on the system and in case they make errors, to help them to easy recovery; User satisfaction: the measure of how pleasant the user finds the system [17]. Usability of an on-line register is its ease of use which includes content, navigation, design, aesthetics and interactivity. Usability is a crucial factor in ensuring that the website is well patronised [18]. If a website is not usable, users will conclude that it is not worth their time and will not visit and use it frequently. The need to carry out a usability evaluation is self-evident, because unless a register is easily usable, access to clinical trial information is obstructed and users will spend more time learning how to use the site than learning from it, or may not even frequent the site at all [18].

**Objective**: To compare access to the information contained in, and the usability of, the SANCTR with five international on-line clinical trials registers.

## Methods

**Searchability**: Three search engines, namely Google, Yahoo! Search and MSN Search (Bing) were used to identify access to SANCTR and the other registers, using the keywords “South African Clinical Trials”.

**Content comparison of on-line registers**: The five international registers were chosen for comparison with SANCTR based on their high ranking when subjected to an on-line search for information on South African clinical trials: The International Clinical Trials Registry Platform (ICTRP), ClinicalTrials.gov, the International Standard Randomised Controlled Trial Number (ISRTN), the Pan African Clinical Trials Register (PACTR) and the European Union Clinical Trials Register (EUCTR). The registers were investigated for information on trials conducted in South Africa using a standardised data extraction form which stipulated 24 data items (Table 1). The form was adapted from the clinical trials registration data set of the WHO [19]. The period covered was from 1 December, 2005 to 1 December, 2015.

**Table 1:** Clinical trial data items

****	Clinical trial data items
**1**	Total number of clinical trials on register
**2**	Number of South African clinical trials on register
**3**	Availability of trial identifying number
**4**	Date of registration in primary register
**5**	Study start date
**6**	Estimated primary completion date
**7**	Title of study
**8**	Summary of study
**9**	Study type
**10**	Study phase
**11**	Health condition(s) or problem(s) studied
**12**	Intervention(s)
**13**	Key inclusion and exclusion criteria
**14**	Primary outcome(s)
**15**	Key secondary outcome(s)
**16**	Target sample size
**17**	Recruitment status
**18**	Country/Countries of recruitment
**19**	Source(s) of monetary or material support
**20**	Primary sponsor
**21**	Secondary sponsor(s)
**22**	Contact details
**23**	"Search" Field Present
**24**	"Advanced Search" Field Present Primary Outcome(s)

**Expert heuristic evaluation**: The usability of the various on-line registers was determined through an expert heuristic evaluation using a questionnaire adapted from the five key usability factors defined by Nielsen [17,18]. Expert heuristic evaluation in this case involved the inspection of the on-line registers using 10 purposively selected experts analysing the website against a list of recognised usability principles [20]. Ten “experts” were purposively chosen from the Department of Pharmacy staff and postgraduate students at Sefako Makgatho Health Sciences University (SMU) with at least three years' internet experience and some knowledge of clinical trials and medicines. The experts were purposively chosen due to their specialist knowledge of medicines in general and clinical trials in particular, coupled with sufficient internet experience deemed to give satisfactory responses to the usability of the online registers. Invitations to participate in the study were sent out via email. The first 10 “experts” who gave consent to participate and respond to the questionnaire were included in the study. The questionnaire required participants to rate their user experience using a five point Likert scale. User experience on the various websites was rated as 1 (Terrible), 2 (Bad), 3 (Average), 4 (Good), 5 (Excellent). In addition, participants were given a task to perform on the various registers to determine “Efficiency, Memorability and Errors”. Using the “Search” option on each website, participants were asked to perform a search based on the key phrase “interventional study for a new drug for diabetes in South Africa” and to report on their experience with each site.

**Ethical considerations**: Ethical clearance for the study was obtained from the Medunsa Research Ethics Committee of the University of Limpopo (MREC/H/228/2014 as amended) before commencement of the data collection.

**Data analysis**: “Ranking”, when carrying out the searchability of the on-line registers, refers to the order with which each of the possible choices appeared on the search engine. The websites were noted and recorded as they appeared on the search engines. Data obtained from the content comparison and the expert heuristic evaluation were transposed into a Microsoft Excel-spreadsheet. The results for each response from the 10 experts on each of the heuristic factors were tabulated. Statistical analysis was carried out using SAS (SAS Institute Inc, Cary, NC, USA), Release 9.4. A mean rating (1-5) was determined for each heuristic factor and an analysis of variance (ANOVA) was performed for comparison of the websites based on the mean values of the heuristic factors were carried out in order to determine the “best” clinical trials register.

**Patient involvement**: No patients/service users/carers/lay people were involved in the design and in the recruitment to and conduct of this study. Neither was the development of outcome measures informed by patients' priorities, experience and preferences. Furthermore, the development of the research question and outcome measures was not directly informed by patients' priorities, experience and preferences.

## Results

**Searchability**: The on-line search using the keywords “South Africa Clinical Trials” through the search engines Google, Yahoo! Search and Bing identified the SANCTR website as having the highest ranking amongst the registers. The PACTR was ranked second, followed by ClinicalTrials.gov, ICTRP, the EU register and ICRTN.

**Figure 1:**
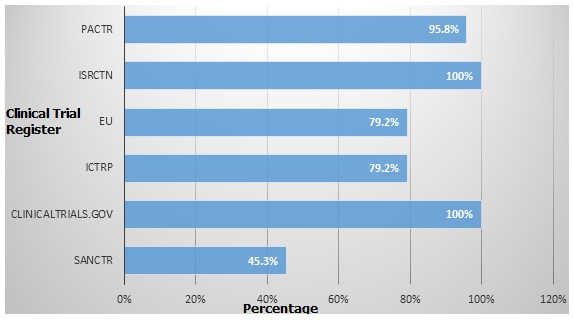
Percentage compliance of clinical trial registers with 24 data items

**Content comparison of online registers**: Table 2 Presents data which show that the WHO's ICTRP registered the highest number of clinical trials carried out in South Africa (2 599), followed by 2 260 registered in the ClinicalTrials.gov register and 2 196 in the SANCTR. The reasons for the discrepancies are unknown but the data showed that SANCTR had 64 fewer South African trials registered than ClinicalTrials.gov and 403 fewer than ICTRP. The other registers had fewer trials registered for South Africa than the SANCTR.Table 2 further shows that there is a lack of standardisation in the registers with each having its own numbering system. The websites ClinicalTrials.gov and ISRCTN provided greater overall information per clinical trial registered and had information on all 24 (100%) clinical trial data items. The SANCTR provided the least information, with only 11 out of the 24 data items being listed (45.3%). The PACTR had information on 23 of the 24 data items (95.8%). The WHO and EU Registers both contained 19 of the 24 data items (79.2%) (Figure 1). Information such as primary outcome, primary sponsor, secondary sponsor, sample size, study type and study phase were missing from the SANCTR. In addition to the clinical trial data items developed for this study, the websites ClinicalTrials.gov and ISRCTN provided information on the results of the clinical trial; date of intention to publish; publication identification for the results if they were published; changes to the clinical trial and a list of complete historical versions of the reports and/or publications from the study.

**Table 2:** Number of clinical trials recorded in the on-line registers as on 1 December 2015

Register	Total number of clinical trials on register	Number of South African clinical trials on register	Primary Registry and Trial Identifying Number System
ClinicalTrials.gov	209 474	2 260	NCT Number
Pan African Clinical Trials Register	658	174	PACTR
SANCTR	2 196	2 196	SANCTR Number
The European Union (EU) Clinical Trials Register	27 412	978	EudraCT Number
The International Clinical Trials Registry Platform (ICTRP) WHO	306 313	2 599	CTRI Number/ UTN Number
The International Standard Randomised Controlled Trial Number (ISRCTN) BioMed Central	14 387	149	ISRCTN

**Expert heuristic evaluation**: Table 3 Shows the results of the expert heuristic evaluation, presented as the mean scores for the 10 experts, rated by eight aspects of usability. The highest score for each criterion is marked in red. ClinicalTrials.gov had the highest mean score and the highest score in six criteria of the nine rated. Statistical analysis (ANOVA) showed that the scores for ClinicalTrials.gov differed widely from those for PACTR and SANCTR. The results for the other registers did not differ much. Amongst the websites surveyed, the criterion “Coherence of the website” scored higher than the other criteria assessed. The data for this measure for the SANCTR and PACTR showed the greatest difference from the mean for the nine items. The overall impression of these two registers was worse than the mean of the ratings for the other measures.Table 3shows the mean score of the usability factors (mean values) per clinical trial register. For each of the six clinical trial registers the mean values for the 9 heuristic factors passed a normality test which allowed an ANOVA for comparison of the websites based on the mean values, which was statistically significant (F test, p=0.009). This step was followed by pair wise comparisons (t tests) of the websites, showing that the overall mean for ClinicalTrials.gov was significantly greater than those of ICTPR (p=0.035), EUCTR (p=0.027), PACTR (p=0.003) and SANCTR (p=0.001). The mean for ISRCTN was significantly greater than the means for PACTR (p=0.043) and for SANCTR (p=0.014).

**Table 3:** :user experience of six on-line registries (n = 10)

Usability criteria		Mean user experience rating* (n=10)						
		SANCTR	Clinical Trials.gov	ICTRP	EUCTR	ISRCTN	PACTR	Average
Layout and design of the on-line registers	Website information	3.58	4.00	3.75	3.67	4.42	3.75	3.86
	Coherence of the website	4.25	4.34	4.25	4.25	4.25	4.25	4.26
	Arrangement of information on the website	3.33	3.75	3.17	3.50	4.00	2.75	3.41
Navigability of the website	Exiting the website	3.33	3.75	3.42	2.92	3.33	3.42	3.36
	Placement of the information on the website	3.17	3.58	3.33	3.25	3.75	2.92	3.33
	Presence of navigation buttons and hyperlinks	2.83	3.92	3.17	2.75	3.25	3.08	3.17
Content features of the online registers	Presence of content and interactive features on the website	2.42	3.58	2.67	2.67	3.17	2.83	2.89
Efficiency, Memorability and Errors in on-line registry	Performance of ‘Search’ option on website	2.00	3.50	2.83	3.08	2.92	2.50	2.81
Overall user-friendliness of the website		2.42	3.83	3.17	3.17	3.33	2.42	3.05
	Mean Score	2.93	3.77	3.26	3.23	3.553	3.04	

* 1 (Terrible); 2 (Bad); 3 (Average); 4 (Good); 5 (Excellent); Highest score for each criterion marked in red

**Other issues raised**: Several of the experts found that the SANCTR and PACTR required login details, however, the option of registering for login was not possible on the websites. It was later discovered that the login feature is only for clinical trial Principal Investigators and staff at the MCC to update clinical trial information. This situation could have the potential of excluding possible users of the site who might be frustrated at not being able to proceed with searching the register. Upon further scrutiny and advice from the researcher, the experts realised that they could access the registers without logging in. One user criticised the ClinicalTrials.gov website as being overloaded with information and hence “too busy”. This aspect could be viewed as an advantage or a disadvantage depending on one's viewpoint. Another user described the information on the PACTR registry as “disjointed”. What was also observed was that the sites catered for different levels of users, from the general lay public to clinicians and advanced, sophisticated clinical trial specialists.

## Discussion

This study revealed that all clinical trial registers are not the same, whether considering overall operation or specifically for a particular country, namely South Africa. It allowed an analysis of the “performance” of the SANCTR against other registers. The SANCTR provided the least information per trial recorded and excluded critical data items such as “sponsor”, “study phase” and study type. The fact that the SANCTR did not have the highest number of South African clinical trials registered, suggests that a number of unregistered clinical trials were possibly being conducted (illegally) in South Africa or that the SANCTR is out of date because some data have not been logged. The SANCTR was also identified as the least “user-friendly”, allowing only a basic search with no options to filter the search result, other than through a list of clinical trials based on disease. Other online registers allowed users to filter and search for trial results according to the various data items such as “Country Location”, “Sponsor”, “Key Inclusion and Exclusion Criteria” and “Clinical Trial Number”. Despite the WHO recommended data set for clinical trial registers 7, there is a lack of standardisation with each register having its own clinical trial numbering system. This lack of standardisation is compounded by the lack of some critical information from the WHO data set.

Information such as type of study, phase of testing, condition treated, and results of the study (including where and when these results were published) would provide useful additional information to users of the various on-line registers. It is also worth mentioning that the same trial listed on several registers varied in the amount of information available on each. It would be ideal to have the SANCTR included in the WHO clinical trials register network so as to experience the benefits of a global network. These include access to a forum to exchange information and working together to establish best practice for clinical trial registration. The SANCTR should be the gold standard for information about clinical trials carried out in South Africa, but it was found lacking in many features. Essential items such as primary outcome, primary sponsor, secondary sponsor, sample size, study type and study phase were missing from the SANCTR. The study has demonstrated that the SANCTR is inadequate as an information source for South African clinical trials. It did not have the highest number of South African clinical trials registered and covered only 11 of the 24 data items investigated. In addition, the website was not user-friendly. An interrogation of the South African MCC to find out why certain data items are excluded from the SANCTR was not carried out as well as to find out why the MCC does not prioritise inclusion of the SANCTR in the WHO clinical trials register network given the value of South Africa as a clinical trials location in the global sphere. Furthermore, the study depended largely on the reliability of the data in the online repositories and assumed that the data were complete and accurate. The limitations of this study could form the basis of future studies on this subject.

## Conclusion

This study demonstrated that the SANCTR is inadequate as an information source for South African clinical trials. This study puts forward a case for reform to the regulatory authority (currently the MCC) as it takes on a new structure and working arrangements as the South African Health Products Regulatory Authority (SAHPRA) to improve the SANCTR to be more user-friendly and to contain more complete information. All trials carried out in South Africa must be registered in the SANCTR, complying with at least the 20 data items required by the WHO. This step will help to ensure that the SANCTR follows global trends which aim to offer as much information as possible to all those involved in, or requiring access to, clinical trial on-line registers, as well as promoting transparency for clinical data, a major current focus for good practice.

### What is known about this topic

- Heuristic evaluations are the gold-standard for usability studies;
- There is need for harmonisation of clinical trial registers.

### What this study adds

- This is the foremost study (documented) that aims to assess the content and usability of the clinical trials registers;
- This study highlights the illustrates how complex the clinical trial registration can be and how this can be eased by making the registers more useable;
- A possible important improvement for all registers (which is sometimes reported in clinicaltrials.gov) would be that all unique registration numbers of the various registers are reported and indexed as search terms, in other clinical trial registers; for example, one could identify in SANCTR search portal a trial also registered in clinicaltrials.gov by searching for the clinicaltrials.gov number.

## Competing interests

The authors declare no competing interest.
